# Impact of the first wave of COVID-19 on outcomes following emergency admissions for common acute surgical conditions: analysis of a national database in England

**DOI:** 10.1093/bjs/znac233

**Published:** 2022-07-27

**Authors:** Andrew Hutchings, Ramani Moonesinghe, Silvia Moler Zapata, David Cromwell, Geoff Bellingan, Ravinder Vohra, Susan Moug, Neil Smart, Robert Hinchliffe, Richard Grieve

**Affiliations:** Department of Health Services Research and Policy, London School of Hygiene and Tropical Medicine, London, UK; Department for Targeted Intervention, Division of Surgery and Interventional Science, University College London, NHS foundation Trust, London, UK; Department of Health Services Research and Policy, London School of Hygiene and Tropical Medicine, London, UK; Department of Health Services Research and Policy, London School of Hygiene and Tropical Medicine, London, UK; Clinical Effectiveness Unit, Royal College of Surgeons of England, London, UK; Department for Targeted Intervention, Division of Surgery and Interventional Science, University College London, NHS foundation Trust, London, UK; Trent Oesophago-Gastric Unit, City Campus, Nottingham University Hospitals NHS Trust, Nottingham, UK; Department of Colorectal Surgery, Royal Alexandra Hospital, Paisley, UK; College of Medicine and Health, University of Exeter, Exeter, UK; Bristol Surgical Trials Centre, University of Bristol, Bristol, UK; Department of Health Services Research and Policy, London School of Hygiene and Tropical Medicine, London, UK

## Abstract

**Background:**

This study assessed the impact of the first COVID-19 wave in England on outcomes for acute appendicitis, gallstone disease, intestinal obstruction, diverticular disease, and abdominal wall hernia.

**Methods:**

Emergency surgical admissions for patients aged 18 years and older to 124 NHS Trust hospitals between January and June in 2019 and 2020 were extracted from Hospital Episode Statistics. The risk of 90-day mortality after admission during weeks 11–19 in 2020 (national lockdown) and 2019 (pre-COVID-19) was estimated using multilevel logistic regression with case-mix adjustment. The primary outcome was all-cause mortality at 90 days.

**Results:**

There were 12 231 emergency admissions and 564 deaths within 90 days during weeks 11–19 in 2020, compared with 18 428 admissions and 542 deaths in the same interval in 2019. Overall, 90-day mortality was higher in 2020 *versus* 2019, with an adjusted OR of 1.95 (95 per cent c.i. 0.78 to 4.89) for appendicitis, 2.66 (1.81 to 3.92) for gallstone disease, 1.99 (1.44 to 2.74) for diverticular disease, 1.70 (1.13 to 2.55) for hernia, and 1.22 (1.01 to 1.47) for intestinal obstruction. After emergency surgery, 90-day mortality was higher in 2020 *versus* 2019 for gallstone disease (OR 3.37, 1.26 to 9.02), diverticular disease (OR 2.35, 1.16 to 4.73), and hernia (OR 2.34, 1.23 to 4.45). For intestinal obstruction, the corresponding OR was 0.91 (0.59 to 1.41). For admissions not leading to emergency surgery, mortality was higher in 2020 *versus* 2019 for gallstone disease (OR 2.55, 1.67 to 3.88), diverticular disease (1.90, 1.32 to 2.73), and intestinal obstruction (OR 1.30, 1.06 to 1.60).

**Conclusion:**

Emergency admission was reduced during the first lockdown in England and this was associated with higher 90-day mortality.

## Teaser

This study compared outcomes after emergency admission during the first wave of COVID-19 in England, with corresponding admissions during 2019, for five common acute surgical conditions. For patients admitted as an emergency during the first national lockdown who did not have emergency surgery, 90-day mortality was higher for some acute conditions, for example acute gallstone disease. Policymakers should maintain appropriate acute surgical care to mitigate the collateral impacts of a crisis on patients’ health.

## Introduction

Emergency general surgery provision is associated with high levels of morbidity, mortality, and resource use^1,2^. The COVID-19 pandemic has had a major impact on surgical provision worldwide as ventilators, hospital space, and personnel were redeployed^3^, and international guidelines increased thresholds for emergency surgery^4^. In the National Health Service (NHS) in England, about one-third of the expected surgical procedures in 2020 were cancelled or postponed^5^. For healthcare commissioners to improve care provision during COVID-19 and future pandemics, evidence is required on the collateral (indirect) effects of delaying or cancelling surgery^6–8^.

During the first wave of the COVID-19 pandemic in England, excess deaths were among the highest in the world^9^, and the large collateral effects may reflect the reduced capacity for acute^10^ and critical^11^ care. The National Emergency Laparotomy Audit^12^, and other studies^13,14^, have reported excess mortality for patients who had emergency surgery and were COVID-19-positive, but lower than expected rates of mortality for those without a COVID-19 diagnosis^12^. There is little evidence regarding outcomes for acute surgical conditions during the COVID-19 period among those who did not have emergency surgery. For patients with confirmed acute appendicitis, first-line antibiotic therapy during the first wave of COVID-19 was associated with improved outcomes compared with emergency surgery^15^. More generally, however, it is unknown whether changes to acute surgical provision during the COVID-19 pandemic, including reduced rates of emergency surgery, had an impact on patients’ health.

This study aimed to compare outcomes after emergency admission during the first wave of COVID-19 in England with corresponding admissions during 2019, for five common acute surgical conditions, namely acute appendicitis, acute symptomatic gallstone disease, intestinal obstruction (small or large bowel), symptomatic diverticular disease, and abdominal wall hernia.

## Methods

### Overview

The ESORT-C19 (Emergency Surgery or Not, COVID-19) study used Hospital Episodes Statistics (HES) data for England to define patient emergency admissions to 124 NHS acute hospital Trusts for five acute surgical conditions^16^. The time intervals of interest were pre-COVID-19 and the first wave of COVID-19. The overall first wave period was defined as the interval from 1 January until 30 June 2020, the last date for which the requisite data were available, with particular interest in weeks 11–19 (11 March to 12 May 2020), which included the first national lockdown in England. The pre-COVID-19 period was defined as the interval from 1 January to 30 June 2019, also with a focus on weeks 11–19. The study considered the impact of the first wave of COVID-19 on overall outcomes, and separately for patients who did and who did not have emergency surgery.

The ESORT-C19 study was approved by the London School of Hygiene and Tropical Medicine ethics committee (ethics reference number 25232). The study involved the secondary analyses of existing pseudonymized data and did not require UK National Ethics Committee approval.

### Patient and public involvement

The design and proposed analysis was informed by a patient and public advisory group during two online workshops held in July 2020. This group will reconvene to discuss the study findings, and co-produce a lay summary that will be made available on the ESORT study website (https://www.lshtm.ac.uk/research/centres-projects-groups/esort).

### Study population

The definitions of the study population and of emergency surgery were those used in the ESORT study^17,18^. For patients aged 18 years old or more, hospital admissions were eligible if a finished consultant episode within the admission met all the following criteria: occurred between 1 January and 30 June in either 2019 or 2020; included a main diagnosis with an ICD-10 diagnosis code (*Table S1*) judged relevant according to the consensus of a clinical panel^19^; was within an emergency admission through the emergency department, or from a primary care referral; was under a consultant general surgeon, subspecialty general surgeon, or any other surgeon working in the general surgery specialty; and was the first or second episode within the admission. For the intestinal obstruction cohort, a relevant diagnosis could appear in the second diagnosis field if the main diagnosis was colorectal cancer. Admissions for which there was a previous emergency admission with a relevant diagnosis in the previous 12 months were excluded.

### Patient-level covariables

The following patient characteristics were extracted from HES data at admission: age (years), sex, ethnicity, Index of Multiple Deprivation (IMD), diagnostic subcategories, and COVID-19-positive status. The Charlson Co-morbidity Index (CCI)^20^ and Secondary Care Administrative Records Frailty (SCARF) index^21^ were derived for all patients. The SCARF index is based on the accumulation of deficits across a number of domains. It uses ICD-10 codes to define 32 deficits that cover functional impairment, geriatric syndromes, problems with nutrition, cognition, mood, and medical co-morbidities, and was developed and validated in a surgical cohort. SCARF uses four categories (fit, mild, moderate or severe frailty), with severe frailty defined by the presence of six or more deficits. COVID-19 was defined as the presence of an ICD-10 diagnosis code of U071 (laboratory-confirmed COVID-19 diagnosis) or U072 (clinical diagnosis without laboratory confirmation) during the index admission, or within any hospital admission up to 90 days.

### Definition of emergency surgery

Emergency surgery was defined from a list of OPCS codes (*Table S2*), and within a maximum time window, of 3 days (hernia), 7 days (appendicitis, gallstone disease, intestinal obstruction), or any time during the emergency admission (diverticular disease), according to the consensus of a clinical panel^19^.

### Process of care

Information from the adult critical care data linked to HES defined time spent in dedicated critical care units as level 2 (high-dependency unit) or level 3 (ICU)^22^. The process-of-care measures included: number of emergency admissions, proportion of emergency surgery, time to emergency surgery, percentage of patients transferred to critical care, mean duration of stay during the index admission, and percentage of elective (within 90 days) and emergency (within 30 or 90 days) readmissions. Process-of-care measures are reported overall, and stratified by whether or not patients had emergency surgery.

### Outcomes

The primary outcome measure was all-cause mortality at 90 days. The secondary outcome was total duration of stay in hospital, including all readmissions up to 90 days. The date of death from linkage to the Office for National Statistics death records, and HES data on the total duration of hospitalization over the 90-day interval are reported. Complete follow-up data were available for all patients.

### Statistical analysis

The study reported trends in the weekly number of emergency admissions for the overall first wave and pre-COVID-19 intervals. Descriptive statistics are presented for patient characteristics, process of care, and outcome measures for weeks 11–19 in 2020 *versus* 2019, overall, and stratified by whether or not patients had emergency surgery. Time to all-cause death is reported using Kaplan–Meier survival curves, stratified by whether or not patients had emergency surgery.

The aim of the statistical analyses was to estimate the association between emergency admission during the first wave of COVID-19 and each outcome, after adjusting for the available case-mix measures. Comparisons of the same time intervals between 2020 and 2019, in particular weeks 11–19, were intended to allow for seasonality and unmeasured prognostic variables that differ over time. Multilevel regression models were developed that included time interval, age (years and years-squared), sex, ethnicity (white, non-white, and unknown), diagnostic subcategories, IMD (quintiles), CCI index and SCARF index as independent variables, and all-cause mortality (binary) and duration of hospital stay (continuous) as dependent variables. Complete-case analysis was performed, with the exception of including an unknown category for ethnicity. The unit of analysis was the emergency admission. The multilevel models included random intercepts for NHS hospital Trusts to allow for clustering, fixed effects for teaching status, and interaction terms of emergency surgery or not, by time interval. The models estimated case mix-adjusted OR, with 95 per cent confidence interval, of all-cause mortality (logistic regression) and mean, with 95 per cent confidence interval, difference in duration of hospital stay (generalized linear models, γ distribution with log links)^23^, for each first wave *versus* pre-COVID-19 interval (weeks 1–10, 11–19, and 20–26), overall and stratified by emergency surgery or not.

Forest plots were used to report the case mix-adjusted ORs for 90-day all-cause mortality for each condition following emergency surgery or not, for 2020 *versus* 2019. All eligible admissions were included, irrespective of COVID-19 status. Admissions with a COVID-19 diagnosis recorded within 90 days were excluded from a sensitivity analysis.

## Results

### Overall cohort

**Fig. 1:**
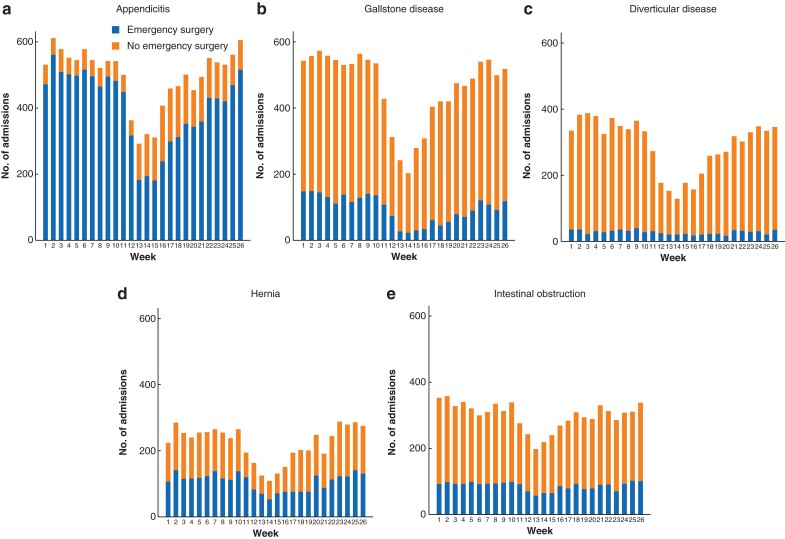
Weekly emergency admissions in weeks 1–26 of 2020 and numbers in receipt of emergency surgery **a** Appendicitis, **b** gallstone disease, **c** diverticular disease, **d** hernia, and **e** intestinal obstruction.

The eligibility of patients and reasons for exclusion are reported in *Table S3*. Compared with 2019, during weeks 11–19 in 2020 the numbers of emergency admissions were reduced by 27.2 per cent (from 4975 to 3620) for appendicitis, 38.0 per cent (4864 to 3016) for gallstone disease, 46.5 per cent (3354 to 1793) for diverticular disease, 39.2 per cent (2418 to 1470) for hernia, and 17.2 per cent (2817 to 2332) for intestinal obstruction (*Fig. 1*). Among the overall cohorts of patients admitted as an emergency, the evaluated characteristics of those admitted during weeks 11–19 in 2020 were similar to those of patients admitted in the same interval in 2019 (*Table 1*). The proportion of patients who had a COVID-19 diagnosis within the index admission in weeks 11–19 of 2020 ranged from 1.5 per cent (gallstone disease) to 2.9 per cent (intestinal obstruction). Other case-mix measures were similar for weeks 11–19 in 2020 *versus* 2019 (*Table 1*).

**Table 1:** Patient characteristics of emergency admissions in weeks 11–19 in 2019 and 2020 for five acute conditions, overall cohorts

	Appendicitis		Gallstone disease		Diverticular disease		Hernia		Intestinal obstruction	
	2019 (*n* = 4975)	2020 (*n* = 3620)	2019 (*n* = 4864)	2020 (*n* = 3016)	2019 (*n* = 3354)	2020 (*n* = 1793)	2019 (*n* = 2418)	2020 (*n* = 1470)	2019 (*n* = 2817)	2020 (*n* = 2332)
**Mean age (years)**	40.8	41.3	55.9	55.9	63.0	62.9	63.1	64.1	68.2	68.0
**Sex (% F)**	47.6	45.6	66.0	64.0	60.2	55.5	37.7	35.2	53.0	53.5
**CCI score (% of patients)**										
0	81.0	81.5	59. 8	61.1	59. 1	58.2	58.0	58.1	48.4	48.1
1	16.1	15.8	28.2	27.8	27.9	29.7	29.4	28.8	34.0	33.0
2	2.4	2.3	9.8	9.0	10.3	9.5	10.2	10.7	13.5	14.7
≥ 3	0.5	0.4	2.2	2.1	2.7	2.6	2.4	2.4	4.1	4.3
**SCARF index (% of patients)**										
Fit	77.9	77.7	53.6	54.4	48.7	49.4	48.9	48.0	38.4	34.8
Mild frailty	18.3	17.4	29.8	29.5	32.1	29.9	30.3	31.4	32.0	34.3
Moderate frailty	2.8	3 .6	12.3	11.2	12.6	12.8	13.6	12.4	17.9	19.6
Severe frailty	1.0	1.3	4.3	4.9	6.6	7.9	7.2	8.2	11.7	11.3
**COVID-19 diagnosis**										
During index admission	–	73 (2.0)	–	46 (1.5)	–	50 (2.8)	–	29 (2.0)	–	67 (2.9)
**Emergency surgery**	4479 (90.0)	2525 (69.8)	1167 (24.0)	458 (15.2)	295 (8.8)	206 (11.5)	1260 (52.1)	701 (47.7)	821 (29.1)	684 (29.3)

Values are *n* (%) unless otherwise indicated, CCI, Charlson Co-morbidity Index; SCARF, Secondary Care Administrative Records Frailty.

A lower proportion of patients with acute appendicitis (69.8 *versus* 90.0 per cent), gallstone disease (15.2 *versus* 24.0 per cent), and hernia (47.7 *versus* 52.1 per cent) had emergency surgery during weeks 11–19 in 2020 compared with 2019 (*Fig. 1* and *Table 1*), with reductions across diagnostic subcategories (*Table S4*). By the end of June 2020, the number of emergency admissions and the proportion of patients who had emergency surgery had returned to 2019 levels.

**Fig. 2:**
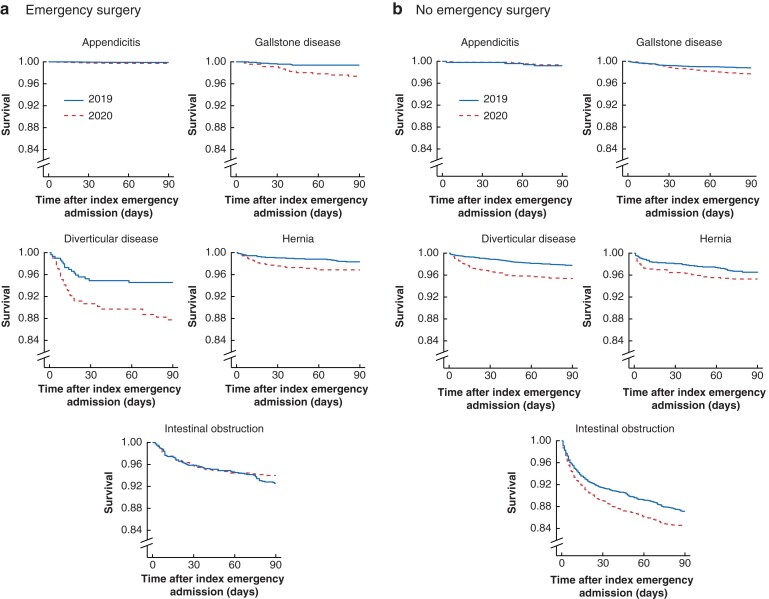
Kaplan–Meier plots comparing 90-day survival in weeks 11–19 of 2020 *versus* 2019 in patients in receipt of emergency surgery or not **a** Emergency surgery and **b** no emergency surgery. The required data on time to death before day 90 were available for each patient, ie, there was no administrative censoring or missing data.

For the overall cohorts, the proportions of transfers to critical care units were lower in 2020 than in 2019 (*Table 2*). The proportions of patients who had emergency readmissions within 90 days were higher in weeks 11–19 in 2020 than in 2019 for those with appendicitis 17.7% (2020) vs 13.1 (2019),gallstone disease 29.3 vs 27.4 and diverticular disease 20.5 vs 18.6 (*Table 2*). For all conditions except appendicitis, the time spent in hospital within 90 days was lower for weeks 11–19 in 2020 compared with 2019; the difference was −0.7 (95 per cent c.i. −0.25 to 0.11) days for appendicitis, −1.02 (−1.32 to −0.72) days for gallstone disease, −1.27 (−1.75 to −0.79) days for diverticular disease, −0.85 (−1.23 to −0.48) days for hernia, and −1.72 (−2.36 to −1.08) days for intestinal obstruction.

**Table 2:** Process of care and outcomes for emergency admissions in weeks 11–19 for 2019 and 2020, overall cohorts

	Appendicitis		Gallstone disease		Diverticular disease		Hernia		Intestinal obstruction	
	2019 (*n* = 4975)	2020 (*n* = 3620)	2019 (*n* = 4864)	2020 (*n* = 3016)	2019 (*n* = 3354)	2020 (*n* = 1793)	2019 (*n* = 2418)	2020 (*n* = 1470)	2019 (*n* = 2817)	2020 (*n* = 2332)
**Index admission**										
Critical care (any) (% of patients)	1.8	1.5	1.8	0.7	5.4	6.2	4.9	3.9	15.2	9.8
Critical care (level 2) (% of patients)	1.7	1.4	1.6	0.7	4.8	5.5	4.5	3.4	14.2	8.7
Critical care (level 3) (% of patients)	0.4	0.5	0.5	0.3	2.5	3.6	1.2	1.2	4.2	3.1
Mean duration of stay (days)	3.2	3.1	4.3	3.5	4.7	4.6	3.0	2.5	8.1	7.0
**Readmission (% of patients)**										
Emergency within 30 days of discharge	10.2	12.9	16.5	16.9	12.0	12.9	12.4	12.8	17.3	18.3
Elective within 90 days	7.9	4.5	29.7	16.5	40.4	11.4	18.9	8.6	16.4	8.8
Emergency within 90 days	13.1	17.7	24.9	28.8	18.6	20.5	20.3	21.2	26.2	26.4
**COVID-19 diagnosis**										
Any inpatient up to 90 days (% of patients)	–	2.5	–	3.4	–	4.6	–	4.0	–	4.9
**Outcomes**										
Mean time spent in hospital at 90 days	5.0	5.0	7.8	6.8	8.2	7.7	6.0	5.1	13.0	11.2
Adjusted difference in 2020 *versus* 2019*†	−0.07 (−0.25, 0.11)		−1.02 (−1.32, −0.72)		−1.27 (−1.75, −0.79)		−0.85 (−1.23, −0.48)		−1.72 (−2.36, −1.08)	
90-day mortality	9 (0.2)	15 (0.4)	51 (1.1)	71(2.4)	89 (2.7)	101 (5.6)	65 (2.7)	62(4.2)	328 (11.6)	315 (13.5)
Adjusted OR in 2020 *versus* 2019*†	1.95 (0.78, 4.89)		2.66 (1.81, 3.92)		1.99 (1.44, 2.74)		1.70 (1.13, 2.55)		1.22 (1.01, 1.47)	

Values are *n* (%) unless otherwise indicated; *values in parentheses are 95 per cent confidence intervals. †Complete-case analysis excluded 162 admissions (appendicitis), 81 (gallstone disease), 46 (diverticular disease), 40 (hernia), and 69 (intestinal obstruction) owing to missing case-mix data.

For all conditions, the all-cause mortality was higher in weeks 11–19 of 2020 compared with 2019. The case mix-adjusted OR was 1.95 (95 per cent c.i. 0.78 to 4.89) for patients with appendicitis, 2.66 (1.81 to 3.92) for those with gallstone disease, 1.99 (1.44 to 2.74) for patients with diverticular disease, 1.70 (1.13 to 2.55) for patients with hernia, and 1.22 (1.01– to 1.47) for those with intestinal obstruction.

### Patients who had emergency surgery

For patients who had emergency surgery, the patient case mix (*Table S5*) and the mean time until surgery (*Table 3*) were similar for weeks 11–19 in 2020 *versus* 2019. The proportions of patients who had critical care (level 2 unit) after emergency surgery for gallstone disease, diverticular disease, hernia or intestinal obstruction were lower for weeks 11–20 in 2020 *versus* 2019. The proportion of emergency readmissions within 90 days was higher in 2020 *versus* 2019 for patients with gallstone disease (26.2 *versus* 17.0 per cent), but similar for the other four conditions. The mean duration of hospital stay by 90 days was longer for gallstone disease but shorter for hernia and intestinal obstruction for patients who had emergency surgery (*Table 3*).

**Table 3:** Process of care and outcomes for emergency admissions in weeks 11–19 for 2019 and 2020, emergency operations

	Appendicitis		Gallstone disease		Diverticular disease		Hernia		Intestinal obstruction	
	2019 (*n* = 4479)	2020 (*n* = 2525)	2019 (*n* = 1167)	2020 (*n* = 458)	2019 (*n* = 295)	2020 (*n* = 206)	2019 (*n* = 1260)	2020 (*n* = 701)	2019 (*n* = 821)	2020 (*n* = 684)
**Index admission**										
Mean time to surgery (days)	0.69	0.74	2.6	2.6	2.7	2.7	0.6	0.6	1.6	1.6
Critical care (any) (% of patients)	1.9	2.1	4.7	2.6	57.6	50.5	8.6	7.1	45.6	29.1
Critical care (level 2) (% of patients)	1.8	1.9	4.2	2.6	51.9	44.2	7.8	6.1	42.9	25.9
Critical care (level 3) (% of patients)	0.4	0.6	1.4	1.5	26.1	30.6	2.0	2.1	12.2	9.6
Mean duration of stay (days)	3.2	3.4	6.0	7.0	18.1	17.2	4.4	3.8	14.2	11.9
**Readmissions (% of patients)**										
Emergency within 30 days of discharge	9.8	11.1	14.4	16.9	17.6	18.0	11.5	10.2	15.7	16.1
Elective within 90 days	6.0	3.2	10.8	9.0	11.9	5.8	7.2	3.6	11.9	8.0
Emergency within 90 days	12.0	13.5	17.0	26.2	21.7	19.9	14.8	14.8	22.2	23.1
**COVID-19 diagnosis**										
Any inpatient up to 90 days (% of patients)	–	1.9	–	4.2	–	12.1	–	4.6	–	4.1
**Outcomes**										
Mean time spent in hospital at 90 days	4.8	5.1	8.4	10.3	21.8	20.5	6.8	5.8	18.1	15.4
Adjusted difference in 2020 *versus* 2019*†	−0.06 (−0.13, 0.25)		1.08 (0.24, 1.91)		−2.41 (−5.33, 0.50)		−0.83 (−1.35, −0.32)		−2.36 (−3.79, −0.93)	
90-day mortality	5 (0.1)	8 (0.3)	7 (0.6)	12 (2.6)	17 (5.8)	27 (13.1)	22 (1.8)	22 (3.1)	62 (7.6)	42 (6.1)
Adjusted OR in 2020 *versus* 2019*†	2.42 (0.71, 8.24)		3.37 (1.26, 9.02)		2.35 (1.16, 4.73)		2.34 (1.23, 4.45)		0.91 (0.59, 1.41)	

Values are *n* (%) unless otherwise indicated; *values in parentheses are 95 per cent confidence intervals. †Complete-case analysis excluded 129 admissions (appendicitis), 14 (gallstone disease), 5 (diverticular disease), 20 (hernia), and 16 (intestinal obstruction) owing to missing case-mix data.

Among patients admitted and having emergency surgery, a higher proportion of those with gallstone disease, diverticular disease or hernia died before 90 days in weeks 11–19 of 2020 compared with 2019; the divergence in mortality occurred before (diverticular disease, hernia) or after (gallstone disease) 30 days (*Fig. 2*). The case mix-adjusted ORs for 90-day mortality for weeks 11–19 of 2020 *versus* 2019 were 3.37 (95 per cent c.i. 1.26 to 9.02) for patients with gallstone disease, 2.35 (1.16 to 4.73) for those with diverticular disease, and 2.34 (1.23 to 4.45) for patients with hernia.

**Fig. 3:**
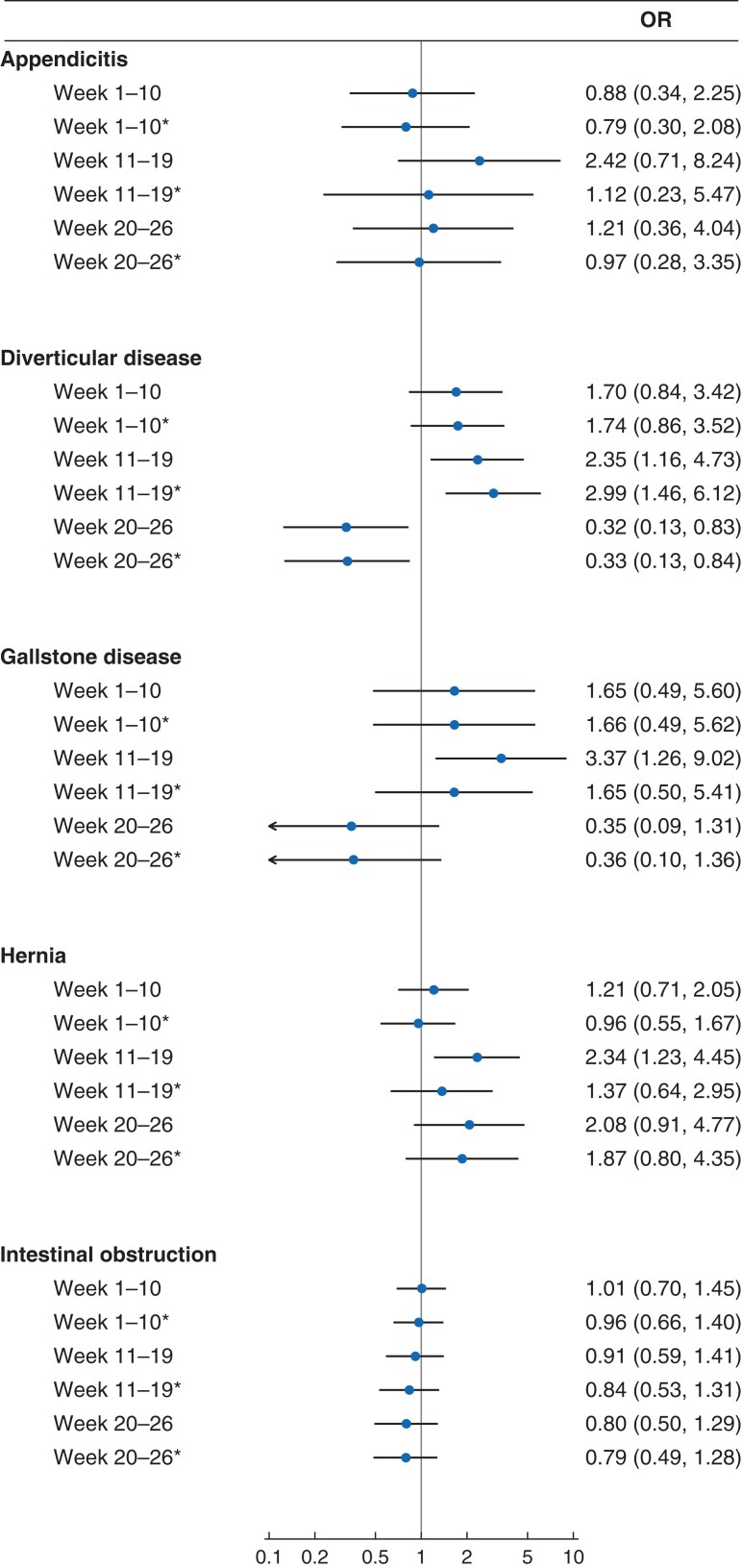
Forest plot showing ORs for 90-day mortality in 2020 *versus* 2019 for patients who had emergency surgery by time interval and inclusion or exclusion of patients diagnosed with COVID ORs are shown with 95 per cent confidence intervals. *Excluding patients with any inpatient COVID-19 diagnosis (ICD-10 codes U071 or U072) within 90 days.

*Figure 3* shows adjusted ORs for 90-day mortality for each interval in 2020 *versus* 2019, for patients who had emergency surgery. For weeks 11–19, the results are presented before (main analysis) and after (sensitivity analysis) exclusion of patients with an inpatient diagnosis of COVID-19 within 90 days. For patients with gallstone disease or hernia who had emergency surgery, the increased mortality was partly attributable to those with an inpatient diagnosis of COVID-19, because the adjusted ORs were reduced to 1.65 (0.50 to 5.41) for gallstone disease and 1.37 (0.64 to 2.95) for hernia, after exclusion of these patients. For patients with diverticular disease, excess mortality remained after excluding those with an inpatient diagnosis of COVID-19, with an estimated OR of 2.99 (1.46 to 6.12).

For emergency admissions during weeks 20–26, there was no evidence of excess mortality during 2020 *versus* 2019, and for those with diverticular disease there was some evidence of reduced mortality for admissions during this period in 2020 (*Fig. 3*).

### Patients who did not have emergency surgery

For patients who did not have emergency surgery, the patient case mix was similar to that for emergency admissions during weeks 11–19 in 2020 *versus* 2019 (*Table S6*). The mean duration of stay during the index emergency admission was lower in weeks 11–19 in 2020 than in 2019 (*Table 4*). The proportion of patients who had an emergency readmission within 30 days was higher in 2020 *versus* 2019 for acute appendicitis (17.1 *versus* 13.9 per cent).

**Table 4:** Process of care and outcomes for emergency admissions in weeks 11–19 for both 2019 and 2020, no emergency operation

	Appendicitis		Gallstone disease		Diverticular disease		Hernia		Intestinal obstruction	
	2019 (*n* = 496)	2020 (*n* = 1095)	2019 (*n* = 3697)	2020 (*n* = 2558)	2019 (*n* = 3059)	2020 (*n* = 1587)	2019 (*n* = 1158)	2020 (*n* = 769)	2019 (*n* = 1996)	2020 (*n* = 1684)
**Index admission**										
Critical care (any) (% of patients)	1.0	0.3	0.8	0.4	0.4	0.4	0.9	0.9	2.7	1.8
Critical care (level 2) (% of patients)	1.0	0.3	0.8	0.3	0.3	0.4	0.9	0.9	2.4	1.6
Critical care (level 3) (% of patients)	0.0	0.1	0.2	0.1	0.2	0.1	0.3	0.3	0.9	0.4
Mean duration of stay (days)	3.5	2.5	3.8	2.9	3.4	3.0	1.6	1.4	5.7	5.0
**Readmissions (%of patients)**										
Emergency within 30 days of discharge	13.9	17.1	17.1	16.9	11.5	12.4	13.3	15.0	17.9	19.2
Elective within 90 days	25.6	7.5	35.7	17.9	43.1	12.2	31.5	13.1	18.2	9.1
Emergency within 90 days	22.6	27.2	27.4	29.3	18.3	20.6	26.2	27.0	27.8	27.8
**Surgery (% of patients)**										
During index admission	1.4	0.5	2.1	0.4	0.0	0.0	2.8	0.9	2.0	1.3
During elective readmission	0.6	0.4	21.8	9.9	0.4	0.4	20.7	6.6	2.6	1.4
During emergency readmission	4.2	6.3	4.5	3.6	1.4	1.6	5.7	5.3	3.3	4.2
**COVID-19 diagnosis**										
Any inpatient up to 90 days (% of patients)	–	3.7	–	3.2	–	3.7	–	3.4	–	5.2
**Outcomes**										
Mean time spent in hospital at 90 days	6.4	4.7	7.6	6.2	6.9	6.0	5.2	4.5	10.9	9.5
Adjusted difference in 2020 *versus* 2019*†	−0.68 (−1.14, −0.21)		−1.36 (−1.69, −1.04)		−1.02 (−1.42, −0.62)		−0.85 −1.37, −0.32)		−1.45 (−2.14, −0.76)	
90-day mortality	4 (0.8)	7 (0.6)	44 (1.2)	59 (2.3)	72 (2.4)	74 (4.7)	43 (3.7)	40 (5.2)	266 (13.3)	273 (16.6)
Adjusted OR in 2020 *versus* 2019*†	1.45 (0.35, 5.95)		2.55 (1.67, 3.88)		1.90 (1.32, 2.73)		1.39 (0.83, 2.32)		1.30 (1.06, 1.60)	

Values are *n* (%) unless otherwise indicated; *values in parentheses are 95 per cent confidence intervals. †Complete-case analysis excluded 33 no emergency surgery admissions (appendicitis), 67 (gallstone disease), 41 (diverticular disease), 20 (hernia), and 53 (intestinal obstruction) owing to missing case-mix data.

The mean duration of hospital stay before 90 days was lower for weeks 11–19 of 2020 *versus* 2019 after adjusting for case mix (*Table 4*). The 90-day mortality rate was higher in 2020 than in 2019 for patients with gallstone disease, diverticular disease, and intestinal obstruction, with adjusted ORs of 2.55 (95 per cent c.i. 1.67 to 3.88), 1.90 (1.32 to 2.73), and 1.30 (1.06 to 1.60) respectively.

**Fig. 4:**
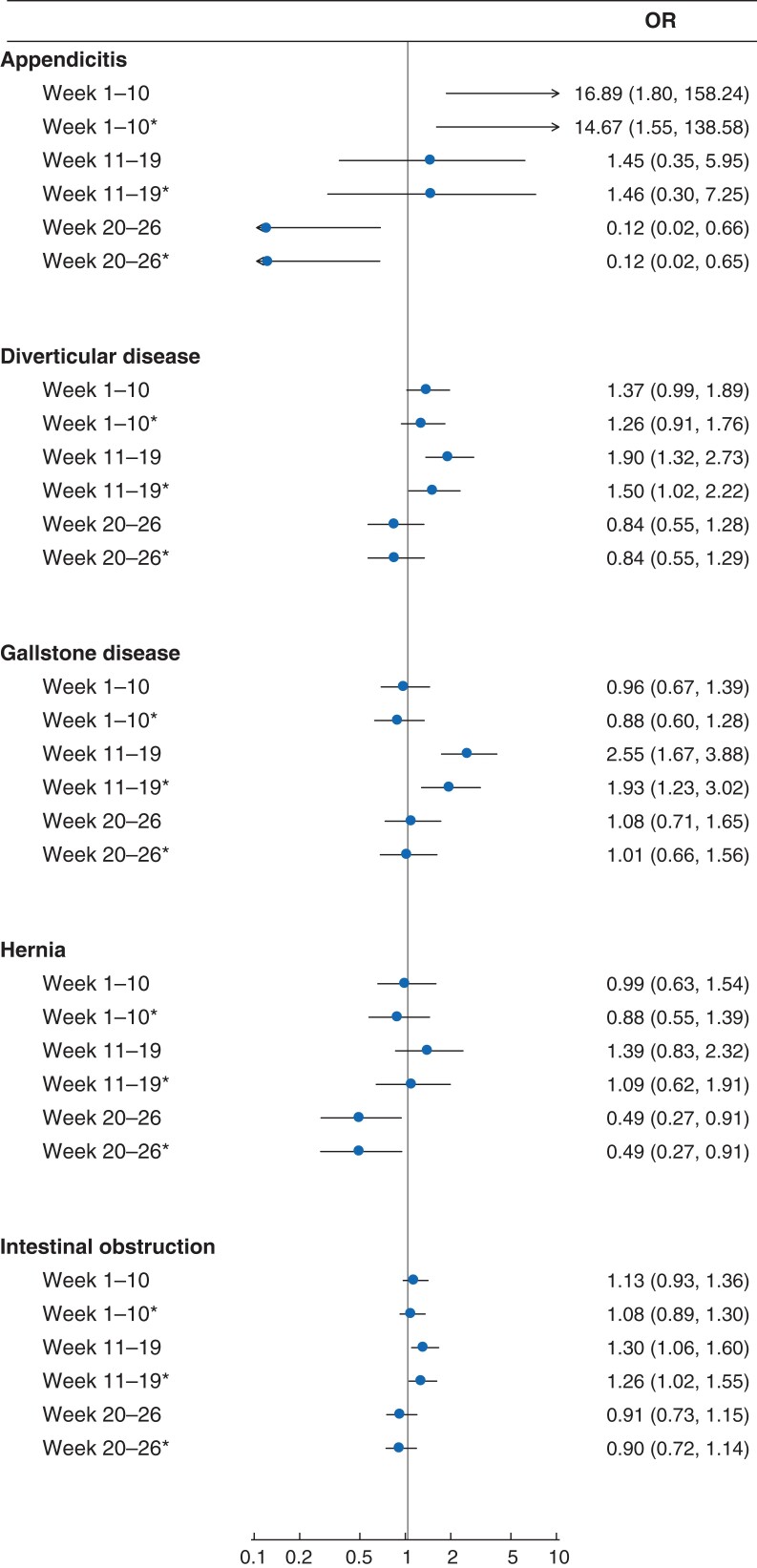
Forest plot showing ORs for 90-day mortality in 2020 *versus* 2019 for patients who did not have emergency surgery by time period and inclusion or exclusion of patients with a COVID diagnosis

*Figure 4* shows the corresponding adjusted ORs for 90-day mortality among patients who did not have emergency surgery, before and after exclusion of those with an inpatient diagnosis of COVID-19. For patients who did not have an inpatient COVID-19 diagnosis, emergency admission during the lockdown period, *versus* 2019, was associated with higher 90-day mortality for patients with gallstone disease (OR 1.93 (1.23 to 3.02), diverticular disease (OR 1.50, 1.02 to 2.72), or intestinal obstruction (OR 1.26, 1.02 to 1.55) for.

ORs are shown with 95 per cent confidence intervals. *Excluding patients with any inpatient COVID-19 diagnosis (ICD-10 codes U071 or U072) within 90 days.

There was no evidence of increased 90-day mortality for weeks 20–26 of 2020 *versus* 2019, for any of the conditions, and some evidence of reduced mortality for patients with hernia (*Fig. 4*).

## Discussion

Overall 90-day mortality was higher for emergency admissions for common acute conditions during the first national lockdown after the onset of the COVID-19 pandemic in England, compared with the same interval in the previous year. Among patients who had emergency surgery, 90-day mortality was higher in 2020 *versus* 2019 for gallstone disease, diverticular disease, and hernia. For admitted patients who did not undergo emergency surgery, mortality was higher in 2020 than 2019 for gallstone disease, diverticular disease, and intestinal obstruction. These findings were evident even after adjustment for measured case mix, including levels of frailty and number of co-morbidities, and after allowing for seasonal differences. It should be recognized that the number of emergency admissions was much reduced during the national lockdown, and the present study design could not adjust for unmeasured differences in patient characteristics, for example acute physiology, in the intervals before and after the onset of COVID-19.

These findings highlight the importance of maintaining and supporting acute surgical services to avoid excess mortality during waves of COVID-19 and other crises. There are several possible explanations for the excess mortality for common acute surgical conditions during the first national lockdown that warrant consideration. Although patients admitted as an emergency in weeks 11–19 in 2020 *versus* 2019 may have been more acutely ill, owing to delayed presentation^24^, strong prognostic health measures, such as age, frailty, and the number of co-morbidities, were similar over time. Moreover, an excess mortality remained after adjusting for case-mix measures that were available in the HES inpatient database. For patients who did not have emergency surgery, the excess death rates in 2020 compared with 2019 could have been explained by selection processes for surgery in 2020, meaning that a higher proportion of high-risk patients did not undergo emergency operations. The case-mix variables measured were, however, similar for patients admitted in 2020 and 2019, both for patients who did and those who did not undergo emergency surgery. This suggests that the excess death rates were not due to differences in selection for surgery. The excess mortality could also have been driven by patients with COVID-19 infection. Indeed, the present study found that, among patients admitted as an emergency for acute hernia or gallstone disease who underwent surgery, the excess mortality was attenuated after excluding those with an inpatient diagnosis of COVID-19. In contrast, for patients with gallstone disease, intestinal obstruction or diverticular disease who did not have emergency surgery, there remained an excess mortality even after excluding those with a COVID-19 inpatient diagnosis.

The higher rates of mortality and emergency readmissions reflect the challenges of maintaining the quality of all aspects of acute surgical service provision during lockdown. For patients who had emergency surgery, the mean time to surgery was similar in 2020 to 2019, in spite of the challenges presented by the reorganization of care pathways and the requirements of infection control processes. Other aspects of acute surgical care, however, differed over this period. In particular, a lower proportion of patients admitted during weeks 11–19 in 2020 were subsequently transferred to critical care beds, reflecting the reduced availability of designated critical care beds for patients without COVID-19^11^. Similarly, staffing numbers were reduced on general medical wards, following redeployment of ward staff to critical care and respiratory wards during surges in COVID-19 activity. Ward nursing numbers have been shown to be inversely proportional to mortality rates across several inpatient settings^25^. Intestinal obstruction had the smallest relative increase in mortality in 2020 *versus* 2019, and also the smallest reduction in numbers admitted and receiving emergency surgery. These findings may be explained by the relative consensus on indications for emergency surgery for intestinal obstruction, amid the lack of alternatives such as antibiotics. Another explanation is that risk of death is higher for intestinal obstruction compared with the other conditions. Thus, any impact of service challenges on mortality in 2020 *versus* 2019 may be similar in absolute terms but smaller in relative terms.

The present study reported lower rates of emergency surgery during the lockdown for conditions apart from intestinal obstruction; these were accompanied by reductions in the mean duration of stay during the primary emergency admission, although followed by higher rates of emergency readmissions. This could reflect the tendency to discharge patients earlier than usual, to reduce pressure on hospital services and in line with patient preferences, but without adequate support for patients and primary care teams. Virtual wards were further developed to enable patients to be discharged earlier, but these services did not generally extend to patients with surgical conditions. For patients with gallstone disease who did not have emergency surgery, the increase in emergency readmissions and excess mortality occurred between 60 and 90 days after admission, reflecting differences in care processes rather than in case mix. Evidence from RCTs has shown that delaying surgery can lead to higher rates of complications and readmissions for patients with acute cholecystitis^26^. During this interval, patients had less access to outpatient and community care, and so those with complications were more likely to be readmitted via emergency departments.

Previous research^12–14,27–29^ found excess mortality during the first wave of COVID-19, but focused on patients who had emergency surgery, or did not separate patients who did and did not have emergency operation. One exception is the COVID-HAREM study^15^, which reported improved outcomes for patients with acute appendicitis who did not have emergency operation compared with those who underwent surgery. In contrast, the present study found an increase in emergency admission rates between 2020 and 2019 for patients who did not have emergency surgery, but similar rates for patients who had surgery. The non-operative strategy was supported by higher rates of imaging (100 per cent) in the COVID-HAREM study^15^ than in the ESORT study (approximately 60 per cent)^18^. This underlines the importance of defining protocols for non-operative strategies, as part of any future strategy to increase thresholds for emergency surgery. The PREDICT study^14^ reported higher excess mortality for an international cohort of emergency surgical admissions in March–May 2020 (adjusted ORs 3.97–4.34), but did not examine excess mortality by condition, or by whether or not patients had emergency surgery.

Here, 90-day mortality during weeks 20–26 was similar in 2020 and 2019, apart from among patients with diverticular disease who had emergency surgery, and those with hernia who did not have emergency surgery. For both these groups, 90-day mortality was lower for the interval after the national lockdown than for the corresponding period in 2019. The most likely explanation for this mortality reduction is mortality displacement: that in 2020 patients who were at high risk died soon after admission during lockdown (weeks 11–19), whereas in 2019 the corresponding group of high-risk patients died later (weeks 20–26).

This study has several important strengths. It used a large national hospital administrative data set to assess the impact of the first wave of COVID-19 on acute surgical provision and outcomes in a publicly funded healthcare system, and so the insights can inform clinical practice in other health systems that also had fewer emergency admissions for acute surgical conditions and lower rates of emergency surgery during the pandemic^27,28^. Almost all adult emergency general surgery activity in England is provided by the 124 hospital Trusts included in this study. The study also included patients who did not have emergency surgery, and was therefore able to take a condition-based approach to provide a broader assessment of the collateral effects of COVID-19 on acute surgical provision^6,14,15,27–29^. The present study also allowed for seasonality and case mix^30^, by including hospital admissions from the same interval in 2019 as control groups, and by adjusting for relevant prognostic measures, such as age, number of co-morbidities, and frailty levels, that were recorded in the HES database.

The study also has limitations. The large reduction in the number of emergency admissions during weeks 11–19 in 2020 *versus* 2019 raises the concern of residual confounding arising from patients missing from the 2020 admissions. It was not possible to adjust for differences in disease severity or patient physiology between patients presenting in 2020 and 2019, and so the estimated associations between the COVID-19 period and all-cause death may have been subjected to selection bias owing to these unmeasured case-mix differences. A COVID-19 diagnosis was not included in the index episode in the case-mix adjustment because the data do not differentiate between a diagnosis occurring before or after surgery. During the first national lockdown, COVID-19 testing was limited, and so the number of COVID-19 cases may have been underestimated, and some of the excess deaths may have been due to undiagnosed COVID-19 infections. Finally, the impact of subsequent waves of COVID-19 infection on outcomes of acute surgical provision was outside the scope of this paper.

Notwithstanding these potential concerns, the finding that the first national lockdown was associated with excess mortality and increased rates of emergency readmissions for patients presenting during the lockdown has wider implications for health service provision. A lockdown reduces the capacity of diagnostic, surgical, and critical care services to diagnose and treat other patients. For example, the first wave of the COVID-19 pandemic in England was associated with substantial reductions in the number of people referred, diagnosed, and treated for colorectal cancer, and this could partly reflect constraints on NHS services^31^. Urgent action is needed to ensure that common acute surgical conditions can be managed with as little disruption to usual care processes as possible.

This study has highlighted that, when preparing for future major incidents, policymakers should ensure that referral and treatment pathways for acute surgical conditions across both primary and secondary care are maintained or strengthened. Immediate audit and research to guide clinical practice for conditions other than just the cause of the incident should be commissioned, and clear public health messaging about how and when to seek help for emergency conditions needs to be provided, to reduce the risk of delayed presentation and consequent worse outcomes. Services and pathways aimed at reducing duration of hospital stay, such as virtual wards^32^, or emergency clinics should be developed outside pandemic conditions to support patients presenting with acute surgical diagnoses, and with the potential to rapidly scale up during emergency surges. Professional bodies for surgery, anaesthesia, and perioperative care should rapidly review evidence as it emerges, and provide guidance to reduce the risk of unnecessary changes in practice, which might be more likely to occur when individual teams have not experienced working in the context of a major incident. These measures may better support clinicians and patients in the event of future emergencies, help reduce variation in practice, and thereby reduce the risk of a future major incident being associated with poor patient outcomes.

## Supplementary Material

znac233_Supplementary_Data
