# Supplementary material for: Impact of the first wave of COVID-19 on outcomes following emergency admissions for common acute surgical conditions: analysis of a national database in England
Source: Br J Surg. 2022 Jul 27;109(10):984–94. doi: 10.1093/bjs/znac233 (PMC9384585; doi:10.1093/bjs/znac233)
Supplement: znac233_Supplementary_Data [file znac233_supplementary_data.docx]

**Supplementary material**

| **Contents** | **Page** |
| --- | --- |
| Table S1: List of eligible International Classification of Diseases 10th Revision (ICD-10) codes and descriptions for defining cohorts | 2 |
| Table S2: List of Office of Population Censuses and Surveys (OPCS-4) codes and descriptions for defining emergency surgery procedures | 3 |
| Table S3: Exclusions of emergency admissions in weeks 11-19 in 2019 and 2020 for five acute conditions | 9 |
| Table S4: Comparison of admissions, receipt of emergency surgery and deaths in weeks 11-19 in 2019 and 2020 | 10 |
| Table S5: Characteristics of patients in receipt of emergency surgery in weeks 11-19 in 2019 and 2020 for five acute conditions | 11 |
| Table S6: Characteristics of patients not in receipt of emergency surgery in weeks 11-19 in 2019 and 2020 for five acute conditions | 12 |

**Table S1: List of eligible International Classification of Diseases 10^th^ Revision (ICD-10) codes** **and descriptions for defining cohorts**

| **Condition** | **ICD-10** | **Description** |
| --- | --- | --- |
| Appendicitis |  |  |
|  | K35.2 | Acute appendicitis with generalized peritonitis |
|  | K35.3 | Acute appendicitis with localized peritonitis |
|  | K35.8 | Acute appendicitis, other and unspecified |
|  | K37 | Unspecified appendicitis |
|  |  |  |
| Diverticular disease | K57.2 | Diverticular disease of large intestine with perforation and abscess |
|  | K57.3 | Diverticular disease of large intestine without perforation or abscess |
|  |  |  |
| Gallstone disease | K80.0 | Calculus of gallbladder with acute cholecystitis |
|  | K80.1 | Calculus of gallbladder with other cholecystitis |
|  | K80.2 | Calculus of gallbladder without cholecystitis |
|  |  |  |
| Hernia | K40.0 | Bilateral inguinal hernia, with obstruction, without gangrene |
|  | K40.1 | Bilateral inguinal hernia, with gangrene |
|  | K40.2 | Bilateral inguinal hernia, without obstruction or gangrene |
|  | K40.3 | Unilateral or unspecified inguinal hernia, with obstruction, without gangrene |
|  | K40.4 | Unilateral or unspecified inguinal hernia, with gangrene |
|  | K40.9 | Unilateral or unspecified inguinal hernia, without obstruction or gangrene |
|  | K41.0 | Bilateral femoral hernia, with obstruction, without gangrene |
|  | K41.1 | Bilateral femoral hernia, with gangrene |
|  | K41.2 | Bilateral femoral hernia, without obstruction or gangrene |
|  | K41.3 | Unilateral or unspecified femoral hernia, with obstruction, without gangrene |
|  | K41.4 | Unilateral or unspecified femoral hernia, with gangrene |
|  | K41.9 | Unilateral or unspecified femoral hernia, without obstruction or gangrene |
|  | K42.0 | Umbilical hernia with obstruction, without gangrene |
|  | K42.1 | Umbilical hernia with gangrene |
|  | K42.9 | Umbilical hernia without obstruction or gangrene |
|  | K43.6 | Other and unspecified ventral hernia with obstruction, without gangrene |
|  | K43.7 | Other and unspecified ventral hernia with gangrene |
|  |  |  |
| Intestinal obstruction | K56.1 | Intussusception |
|  | K56.2 | Volvulus |
|  | K56.3 | Gallstone ileus |
|  | K56.5 | Intestinal adhesions [bands] with obstruction |
|  | K56.6 | Other and unspecified intestinal obstruction |

**Table S2: List of Office of Population Censuses and Surveys (OPCS-4) codes and descriptions for defining emergency surgery procedures**

| **Appendicitis** |
| --- |
| G694: Ileectomy and anastomosis of ileum to colon |
| H011: Emergency excision of abnormal appendix and drainage HFQ |
| H012: Emergency excision of abnormal appendix NEC |
| H013: Emergency excision of normal appendix |
| H018: Other specified emergency excision of appendix |
| H019: Unspecified emergency excision of appendix |
| H029: Unspecified other excision of appendix |
| H031: Drainage of abscess of appendix |
| H032: Drainage of appendix NEC |
| H062: Extended right hemicolectomy and anastomosis of ileum to colon |
| H071: Right hemicolectomy and end to end anastomosis of ileum to colon |
| H072: Right hemicolectomy and side to side anastomosis of ileum to transverse colon |
| H073: Right hemicolectomy and anastomosis NEC |
| H074: Right hemicolectomy and ileostomy HFQ |
| H078: Other specified other excision of right hemicolon |
| H079: Unspecified other excision of right hemicolon |
| T342: Open drainage of pelvic abscess |
| T343: Open drainage of abdominal abscess NEC |
| T452: Image controlled percutaneous drainage of pelvic abscess |
| T453: Image controlled percutaneous drainage of abdominal abscess NEC |
| T463: Irrigation of peritoneal cavity |
| **Diverticular disease** |
| G742: Creation of temporary ileostomy |
| G743: Creation of defunctioning ileostomy |
| H053: Total colectomy and ileostomy NEC |
| H062: Extended right hemicolectomy and anastomosis of ileum to colon |
| H064: Extended right hemicolectomy and ileostomy HFQ |
| H071: Right hemicolectomy and end to end anastomosis of ileum to colon |
| H072: Right hemicolectomy and side to side anastomosis of ileum to transverse colon |
| H073: Right hemicolectomy and anastomosis NEC |
| H074: Right hemicolectomy and ileostomy HFQ |
| H091: Left hemicolectomy and end to end anastomosis of colon to rectum |
| H092: Left hemicolectomy and end to end anastomosis of colon to colon |
| H093: Left hemicolectomy and anastomosis NEC |
| H094: Left hemicolectomy and ileostomy HFQ |
| H095: Left hemicolectomy and exteriorisation of bowel NEC |
| H101: Sigmoid colectomy and end to end anastomosis of ileum to rectum |
| H102: Sigmoid colectomy and anastomosis of colon to rectum |
| H103: Sigmoid colectomy and anastomosis NEC |
| H104: Sigmoid colectomy and ileostomy HFQ |
| H105: Sigmoid colectomy and exteriorisation of bowel NEC |
| H108: Other specified excision of sigmoid colon |
| H109: Unspecified excision of sigmoid colon |
| H111: Colectomy and end to end anastomosis of colon to colon NEC |
| H112: Colectomy and side to side anastomosis of ileum to colon NEC |
| H113: Colectomy and anastomosis NEC |
| H114: Colectomy and ileostomy NEC |
| H115: Colectomy and exteriorisation of bowel NEC |
| H121: Excision of diverticulum of colon |
| H151: Loop colostomy |
| H152: End colostomy |
| H158: Other specified other exteriorisation of colon |
| H159: Unspecified other exteriorisation of colon |
| H161: Drainage of colon |
| H298: Other specified subtotal excision of colon |
| H299: Unspecified subtotal excision of colon |
| H333: Anterior resection of rectum and anastomosis of colon to rectum using staples |
| H334: Anterior resection of rectum and anastomosis NEC |
| H335: Rectosigmoidectomy and closure of rectal stump and exteriorisation of bowel |
| H336: Anterior resection of rectum and exteriorisation of bowel |
| T342: Open drainage of pelvic abscess |
| T343: Open drainage of abdominal abscess NEC |
| T348: Other specified open drainage of peritoneum |
| T349: Unspecified open drainage of peritoneum |
| T463: Irrigation of peritoneal cavity |
| T468: Other specified other drainage of peritoneal cavity |
| T469: Unspecified other drainage of peritoneal cavity |
| **Gallstone disease** |
| J181: Total cholecystectomy and excision of surrounding tissue |
| J182: Total cholecystectomy and exploration of common bile duct |
| J183: Total cholecystectomy NEC |
| J184: Partial cholecystectomy and exploration of common bile duct |
| J185: Partial cholecystectomy NEC |
| J188: Other specified excision of gall bladder |
| J189: Unspecified excision of gall bladder |
| J211: Open removal of calculus from gall bladder |
| J212: Drainage of gall bladder |
| J213: Drainage of tissue surrounding gall bladder |
| J241: Percutaneous drainage of gall bladder |
| **Hernia** |
| G762: Open relief of strangulation of ileum |
| G763: Open relief of obstruction of ileum NEC |
| H176: Open relief of obstruction of colon NEC |
| T192: Unilateral herniotomy |
| T199: Unspecified simple excision of inguinal hernial sac |
| T201: Primary repair of inguinal hernia using insert of natural material |
| T202: Primary repair of inguinal hernia using insert of prosthetic material |
| T203: Primary repair of inguinal hernia using sutures |
| T204: Primary repair of inguinal hernia and reduction of sliding hernia |
| T208: Other specified primary repair of inguinal hernia |
| T209: Unspecified primary repair of inguinal hernia |
| T211: Repair of recurrent inguinal hernia using insert of natural material |
| T212: Repair of recurrent inguinal hernia using insert of prosthetic material |
| T213: Repair of recurrent inguinal hernia using sutures |
| T218: Other specified repair of recurrent inguinal hernia |
| T219: Unspecified repair of recurrent inguinal hernia |
| T221: Primary repair of femoral hernia using insert of natural material |
| T222: Primary repair of femoral hernia using insert of prosthetic material |
| T223: Primary repair of femoral hernia using sutures |
| T228: Other specified primary repair of femoral hernia |
| T229: Unspecified primary repair of femoral hernia |
| T231: Repair of recurrent femoral hernia using insert of natural material |
| T232: Repair of recurrent femoral hernia using insert of prosthetic material |
| T233: Repair of recurrent femoral hernia using sutures |
| T239: Unspecified repair of recurrent femoral hernia |
| T241: Repair of umbilical hernia using insert of natural material |
| T242: Repair of umbilical hernia using insert of prosthetic material |
| T243: Repair of umbilical hernia using sutures |
| T248: Other specified primary repair of umbilical hernia |
| T249: Unspecified primary repair of umbilical hernia |
| T251: Primary repair of incisional hernia using insert of natural material |
| T252: Primary repair of incisional hernia using insert of prosthetic material |
| T253: Primary repair of incisional hernia using sutures |
| T258: Other specified primary repair of incisional hernia |
| T259: Unspecified primary repair of incisional hernia |
| T261: Repair of recurrent incisional hernia using insert of natural material |
| T262: Repair of recurrent incisional hernia using insert of prosthetic material |
| T263: Repair of recurrent incisional hernia using sutures |
| T268: Other specified repair of recurrent incisional hernia |
| T269: Unspecified repair of recurrent incisional hernia |
| T271: Repair of ventral hernia using insert of natural material |
| T272: Repair of ventral hernia using insert of prosthetic material |
| T273: Repair of ventral hernia using sutures |
| T274: Removal of prosthetic material from previous repair of ventral hernia |
| T278: Other specified repair of other hernia of abdominal wall |
| T279: Unspecified repair of other hernia of abdominal wall |
| T283: Resuture of previous incision of anterior abdominal wall |
| T288: Other specified other repair of anterior abdominal wall |
| T318: Other specified other operations on anterior abdominal wall |
| T971: Repair of recurrent umbilical hernia using insert of natural material |
| T972: Repair of recurrent umbilical hernia using insert of prosthetic material |
| T973: Repair of recurrent umbilical hernia using sutures |
| T978: Other specified repair of recurrent umbilical hernia |
| T979: Unspecified repair of recurrent umbilical hernia) |
| T981: Repair of recurrent ventral hernia using insert of natural material |
| T982: Repair of recurrent ventral hernia using insert of prosthetic material |
| T983: Repair of recurrent ventral hernia using sutures |
| T989: Unspecified repair of recurrent other hernia of abdominal wall |
| **Intestinal obstruction** |
| G584: Partial jejunectomy and anastomosis of jejunum to ileum |
| G588: Other specified excision of jejunum |
| G589: Unspecified excision of jejunum |
| G591: Excision of lesion of jejunum |
| G601: Creation of jejunostomy |
| G611: Bypass of jejunum by anastomosis of jejunum to jejunum |
| G612: Bypass of jejunum by anastomosis of jejunum to ileum |
| G613: Bypass of jejunum by anastomosis of jejunum to colon |
| G691: Ileectomy and anastomosis of stomach to ileum |
| G692: Ileectomy and anastomosis of duodenum to ileum |
| G693: Ileectomy and anastomosis of ileum to ileum |
| G694: Ileectomy and anastomosis of ileum to colon |
| G698: Other specified excision of ileum |
| G699: Unspecified excision of ileum |
| G702: Excision of lesion of ileum NEC |
| G711: Bypass of ileum by anastomosis of jejunum to ileum |
| G712: Bypass of ileum by anastomosis of ileum to ileum |
| G713: Bypass of ileum by anastomosis of ileum to caecum |
| G714: Bypass of ileum by anastomosis of ileum to transverse colon |
| G715: Bypass of ileum by anastomosis of ileum to colon NEC |
| G718: Other specified bypass of ileum |
| G719: Unspecified bypass of ileum |
| G722: Anastomosis of ileum to transverse colon |
| G723: Anastomosis of ileum to colon NEC |
| G728: Other specified other connection of ileum |
| G734: Resection of ileocolic anastomosis |
| G742: Creation of temporary ileostomy |
| G743: Creation of defunctioning ileostomy |
| G761: Open reduction of intussusception of ileum |
| G762: Open relief of strangulation of ileum |
| G763: Open relief of obstruction of ileum NEC |
| G782: Strictureplasty of ileum |
| G783: Removal of foreign body from ileum |
| H051: Total colectomy and anastomosis of ileum to rectum |
| H053: Total colectomy and ileostomy NEC |
| H061: Extended right hemicolectomy and end to end anastomosis |
| H062: Extended right hemicolectomy and anastomosis of ileum to colon |
| H063: Extended right hemicolectomy and anastomosis NEC |
| H064: Extended right hemicolectomy and ileostomy HFQ |
| H068: Other specified extended excision of right hemicolon |
| H069: Unspecified extended excision of right hemicolon |
| H071: Right hemicolectomy and end to end anastomosis of ileum to colon |
| H072: Right hemicolectomy and side to side anastomosis of ileum to transverse colon |
| H073: Right hemicolectomy and anastomosis NEC |
| H074: Right hemicolectomy and ileostomy HFQ |
| H075: Right hemicolectomy and end to side anastomosis |
| H078: Other specified other excision of right hemicolon |
| H079: Unspecified other excision of right hemicolon |
| H081: Transverse colectomy and end to end anastomosis |
| H082: Transverse colectomy and anastomosis of ileum to colon |
| H083: Transverse colectomy and anastomosis NEC |
| H085: Transverse colectomy and exteriorisation of bowel NEC |
| H091: Left hemicolectomy and end to end anastomosis of colon to rectum |
| H092: Left hemicolectomy and end to end anastomosis of colon to colon |
| H093: Left hemicolectomy and anastomosis NEC |
| H094: Left hemicolectomy and ileostomy HFQ |
| H095: Left hemicolectomy and exteriorisation of bowel NEC |
| H101: Sigmoid colectomy and end to end anastomosis of ileum to rectum |
| H102: Sigmoid colectomy and anastomosis of colon to rectum |
| H103: Sigmoid colectomy and anastomosis NEC |
| H104: Sigmoid colectomy and ileostomy HFQ |
| H105: Sigmoid colectomy and exteriorisation of bowel NEC |
| H109: Unspecified excision of sigmoid colon |
| H111: Colectomy and end to end anastomosis of colon to colon NEC |
| H112: Colectomy and side to side anastomosis of ileum to colon NEC |
| H113: Colectomy and anastomosis NEC |
| H114: Colectomy and ileostomy NEC |
| H115: Colectomy and exteriorisation of bowel NEC |
| H119: Unspecified other excision of colon |
| H122: Excision of lesion of colon NEC |
| H131: Bypass of colon by anastomosis of ileum to colon |
| H138: Other specified bypass of colon |
| H141: Tube caecostomy |
| H149: Unspecified exteriorisation of caecum |
| H151: Loop colostomy |
| H152: End colostomy |
| H158: Other specified other exteriorisation of colon |
| H159: Unspecified other exteriorisation of colon |
| H171: Open reduction of intussusception of colon |
| H172: Open reduction of volvulus of caecum |
| H173: Open reduction of volvulus of sigmoid colon |
| H174: Open reduction of volvulus of colon NEC |
| H176: Open relief of obstruction of colon NEC |
| H194: Open removal of foreign body from colon |
| H298: Other specified subtotal excision of colon |
| H299: Unspecified subtotal excision of colon |
| H333: Anterior resection of rectum and anastomosis of colon to rectum using staples |
| H334: Anterior resection of rectum and anastomosis NEC |
| H335: Rectosigmoidectomy and closure of rectal stump and exteriorisation of bowel |
| H336: Anterior resection of rectum and exteriorisation of bowel |
| Intestinal obstruction |
| T202: Primary repair of inguinal hernia using insert of prosthetic material |
| T203: Primary repair of inguinal hernia using sutures |
| T209: Unspecified primary repair of inguinal hernia |
| T212: Repair of recurrent inguinal hernia using insert of prosthetic material |
| T222: Primary repair of femoral hernia using insert of prosthetic material |
| T223: Primary repair of femoral hernia using sutures |
| T229: Unspecified primary repair of femoral hernia |
| T242: Repair of umbilical hernia using insert of prosthetic material |
| T243: Repair of umbilical hernia using sutures |
| T252: Primary repair of incisional hernia using insert of prosthetic material |
| T253: Primary repair of incisional hernia using sutures |
| T259: Unspecified primary repair of incisional hernia |
| T262: Repair of recurrent incisional hernia using insert of prosthetic material |
| T272: Repair of ventral hernia using insert of prosthetic material |
| T273: Repair of ventral hernia using sutures |
| T278: Other specified repair of other hernia of abdominal wall |
| T279: Unspecified repair of other hernia of abdominal wall |
| T412: Division of band of peritoneum |
| T413: Freeing of adhesions of peritoneum |
| T415: Freeing of extensive adhesions of peritoneum |
| T423: Endoscopic division of adhesions of peritoneum |

**Table S3: Exclusions of emergency admissions in weeks 11-19 in 2019 and 2020 for five acute conditions**

|  | **Appendicitis** | | **Gallstone disease** | | **Diverticular disease** | | **Hernia** | | **Intestinal obstruction** | |
| --- | --- | --- | --- | --- | --- | --- | --- | --- | --- | --- |
|  | 2019 | 2020 | 2019 | 2020 | 2019 | 2020 | 2019 | 2020 | 2019 | 2020 |
| *Episode in an emergency admission with a relevant primary diagnosis*: n* | *5,809* | *4,461* | *7,661* | *4,813* | *4,826* | *2,665* | *3,442* | *2,121* | *5,017* | *4,221* |
|  |  |  |  |  |  |  |  |  |  |  |
| **Exclusion criteria** |  |  |  |  |  |  |  |  |  |  |
| No episode with a consultant surgeon: n (%) | 163 | 147 | 1,185 | 779 | 604 | 387 | 234 | 158 | 683 | 590 |
|  | (2·8) | (3·3) | (15·5) | (16·2) | (12·5) | (14·5) | (6·8) | (7·4) | (13·6) | (14·0) |
| No eligible diagnosis in the first two episodes: n (%) | 37 | 28 | 114 | 72 | 92 | 38 | 42 | 18 | 168 | 77 |
|  | (0·6) | (0·6) | (1·5) | (1·5) | (1·9) | (1·4) | (1·2) | (0·8) | (3·3) | (1·8) |
| Not admitted through A&E or GP: n (%) | 455 | 426 | 584 | 409 | 308 | 178 | 282 | 191 | 271 | 283 |
|  | (7·8) | (9·5) | (7·6) | (8·5) | (6·4) | (6·7) | (8·2) | (9·0) | (5·4) | (6·7) |
| Clinical panel exclusion criteria†: n (%) | 32 | 30 | 0 | 0 | 0 | 0 | 199 | 110 | 265 | 261 |
|  | (0·6) | (0·7) | (0·0) | (0·0) | (0·0) | (0·0) | (5·8) | (5·2) | (5·3) | (6·2) |
| Missing discharge data: n (%) | 9 | 8 | 4 | 1 | 4 | 3 | 3 | 3 | 5 | 4 |
|  | (0·2) | (0·2) | (0·1) | (0·0) | (0·1) | (0·1) | (0·1) | (0·1) | (0·1) | (0·1) |
| Other admission meeting inclusion criteria in previous year: n (%) | 96 | 157 | 881 | 517 | 450 | 257 | 232 | 147 | 708 | 581 |
|  | (1·7) | (3·5) | (11·5) | (10·7) | (9·3) | (9·6) | (6·7) | (6·9) | (14·1) | (13·8) |
| Emergency surgery (ES) prior to index episode: n (%) | 42 | 43 | 16 | 8 | 11 | 7 | 15 | 12 | 17 | 19 |
|  | (0·7) | (1·0) | (0·2) | (0·2) | (0·2) | (0·3) | (0·4) | (0·6) | (0·3) | (0·5) |
| ES in prior admission within 90 days: n (%) | 0 | 2 | 13 | 10 | 3 | 2 | 17 | 11 | 83 | 64 |
|  | (0·0) | (0·0) | (0·2) | (0·2) | (0·1) | (0·1) | (0·5) | (0·5) | (1·7) | (1·5) |
| All exclusions: n (%) | 834 | 841 | 2,797 | 1,796 | 1,472 | 872 | 1,024 | 650 | 2,200 | 1,879 |
|  | (14·4) | (18·9) | (36·5) | (37·3) | (30·5) | (32·7) | (29·8) | (30·6) | (43·9) | (44·5) |
|  |  |  |  |  |  |  |  |  |  |  |
| *Final cohort: n* | *4,975* | *3,620* | *4,864* | *3,016* | *3,354* | *1,793* | *2,418* | *1,470* | *2,817* | *2,332* |

*For intestinal obstruction a relevant diagnosis could appear in the second diagnosis field if the primary diagnosis was colorectal cancer (ICD10 codes C18-C20); †Exclusion criteria were pregnancy or appendiceal cancer for appendicitis, pregnancy, ischaemia or cancer for hernia, colorectal cancer with metastases, gynaecological cancer or ischaemia for intestinal obstruction.

***Table S4: Comparison of admissions, receipt of emergency surgery and deaths in weeks 11-19 in 2019 and 2020***

|  | **Admissions** | | **Emergency surgery** | | **Deaths within 90 days** | |
| --- | --- | --- | --- | --- | --- | --- |
| **Appendicitis** | 2019 | 2020 | 2019 | 2020 | 2019 | 2020 |
| Acute appendicitis with generalized peritonitis | 124 | 82 | 118 | 76 | * | * |
| Acute appendicitis with localized peritonitis | 1,662 | 1,152 | 1,523 | 1,021 | * | * |
| Acute appendicitis, other and unspecified | 2,603 | 1,963 | 2,410 | 1,294 | * | * |
| Unspecified appendicitis | 586 | 423 | 428 | 134 | * | * |
|  |  |  |  |  |  |  |
| **Gallstone disease** |  |  |  |  |  |  |
| Calculus of gallbladder with acute cholecystitis | 2,018 | 1,367 | 652 | 304 | 26 | 42 |
| Calculus of gallbladder with other cholecystitis | 1,359 | 763 | 382 | 113 | 11 | 17 |
| Calculus of gallbladder without cholecystitis | 1,487 | 886 | 133 | 41 | 14 | 12 |
|  |  |  |  |  |  |  |
| **Diverticular disease** |  |  |  |  |  |  |
| Diverticular disease of large intestine with perforation and abscess | 731 | 549 | 253 | 183 | 40 | 72 |
| Diverticular disease of large intestine without perforation or abscess | 2,623 | 1,244 | 42 | 23 | 49 | 29 |
|  |  |  |  |  |  |  |
| **Hernia - site** |  |  |  |  |  |  |
| Inguinal | 1,144 | 705 | 482 | 249 | 34 | 28 |
| Femoral | 288 | 168 | 253 | 147 | 13 | 16 |
| Umbilical | 864 | 511 | 443 | 247 | 11 | 15 |
| Ventral | 122 | 86 | 82 | 58 | * | * |
| **Hernia - bilateral** |  |  |  |  |  |  |
| No | 2,344 | 1,435 | 1,232 | 692 | 60 | 61 |
| Yes | 74 | 35 | 28 | 9 | * | * |
| **Hernia - obstruction/gangrene** |  |  |  |  |  |  |
| None | 1,259 | 726 | 341 | 157 | * | 9 |
| Obstruction | 1,110 | 706 | 874 | 511 | 54 | 48 |
| Gangrene | 49 | 38 | 45 | 33 | * | * |
|  |  |  |  |  |  |  |
| **Intestinal obstruction** |  |  |  |  |  |  |
| Intussusception | 21 | 21 | 13 | 11 | * | * |
| Volvulus | 310 | 245 | 80 | 67 | 37 | 52 |
| Gallstone ileus | 32 | 33 | 15 | 15 | * | * |
| Intestinal adhesions [bands] with obstruction | 993 | 844 | 451 | 373 | 64 | 54 |
| Other and unspecified intestinal obstruction | 1,461 | 1,189 | 262 | 218 | 227 | 204 |

*small numbers suppressed

**Table S5: Characteristics of patients in receipt of emergency surgery in weeks 11-19 in 2019 and 2020 for five acute conditions**

|  | **Appendicitis** | | **Gallstone disease** | | **Diverticular disease** | | **Hernia** | | **Intestinal obstruction** | |
| --- | --- | --- | --- | --- | --- | --- | --- | --- | --- | --- |
|  | *2019*  *n=4,479* | *2020*  *n=2,525* | *2019*  *n=1,167* | *2020*  *n=458* | *2019*  *n=295* | *2020*  *n=206* | *2019*  *n=1,260* | *2020*  *n=701* | *2019*  *n=821* | *2020*  *n=684* |
| **Age: mean years** | 40·0 | 40·9 | 52·8 | 56·8 | 63·3 | 63·6 | 63·8 | 64·7 | 67·4 | 66·6 |
| **Sex: female %** | 47·7 | 44·8 | 68·3 | 60·7 | 56·6 | 57·8 | 43·3 | 42·7 | 56·3 | 58·6 |
|  |  |  |  |  |  |  |  |  |  |  |
| **Charlson comorbidity index: %**  None  One  Two  Three or more | 82·2  15·4  2·0  0·4 | 82·7  15·1  2·0  0·2 | 65·1  25·8  8·0  1·1 | 60·7  28·8  9·2  1·3 | 67·1  23·1  8·5  1·4 | 58·7  31·6  8·7  1·0 | 60·0  30·5  7·7  1·8 | 59·3  29·1  9·3  2·3 | 51·8  32·9  11·8  3·5 | 52·3  32·8  11·0  4·0 |
| **Frailty SCARF index: %**  Fit  Mild frailty  Moderate frailty  Severe frailty | 78·8  17·9  2·5  0·8 | 77·3  18·3  3·5  1·0 | 54·8  31·7  10·1  3·4 | 46·1  34·5  14·4  5·0 | 29·2  40·3  18·6  11·9 | 29·6  33·0  20·9  16·5 | 47·2  32·5  13·4  6·9 | 46·5  32·7  12·1  8·7 | 34·1  31·4  20·0  14·6 | 31·4  38·9  19·9  9·8 |
| **COVID diagnosis** |  |  |  |  |  |  |  |  |  |  |
| Diagnosis in index admission: n (%) | .. | 37  (1·5) | .. | 9  (2·0) | .. | 14  (6·8) | .. | 22  (3·1) | .. | 19  (2·8) |

**Table S6: Characteristics of patients not in receipt of emergency surgery in weeks 11-19 in 2019 and 2020 for five acute conditions**

|  | **Appendicitis** | | **Gallstone disease** | | **Diverticular disease** | | **Hernia** | | **Intestinal obstruction** | |
| --- | --- | --- | --- | --- | --- | --- | --- | --- | --- | --- |
|  | *2019*  *n=496* | *2020*  *n=1,095* | *2019*  *n=3,697* | *2020*  *n=2,558* | *2019*  *n=3,059* | *2020*  *n=1,587* | *2019*  *n=1,158* | *2020*  *n=769* | *2019*  *n=1,996* | *2020*  *n=1,684* |
| **Age: mean years** | 48·7 | 42·2 | 56·8 | 55·8 | 63·0 | 62·8 | 62·4 | 63·5 | 68·5 | 68·6 |
| **Sex: female %** | 47·0 | 47·5 | 65·2 | 64·7 | 60·6 | 55·3 | 31·6 | 28·5 | 51·7 | 51·5 |
|  |  |  |  |  |  |  |  |  |  |  |
| **Charlson comorbidity index: %**  None  One  Two  Three or more | 69·8  22·6  6·1  1·6 | 78·8  17·4  3·1  0·7 | 58·2  28·9  10·3  2·6 | 61·1  27·6  9·0  2·3 | 58·3  28·4  10·5  2·8 | 58·2  29·5  9·6  2·8 | 55·8  28·2  12·9  3·1 | 57·0  28·6  12·0  2·5 | 46·9  34·5  14·2  4·4 | 46·4  33·1  16·2  4·4 |
| **Frailty SCARF index: %**  Fit  Mild frailty  Moderate frailty  Severe frailty | 69·2  21·8  6·1  3·0 | 78·8  15·3  3·8  2·1 | 53·3  29·2  12·9  4·6 | 55·9  28·6  10·6  4·9 | 50·6  31·3  12·1  6·1 | 52·0  29·5  11·8  6·7 | 50·8  28·0  13·8  7·4 | 49·4  30·2  12·6  7·8 | 40·2  32·2  18·1  11·1 | 36·2  32·4  19·5  11·9 |
| **COVID diagnosis** |  |  |  |  |  |  |  |  |  |  |
| Diagnosis in index admission: n (%) | .. | 36  (3·3) | .. | 37  (1·5) | .. | 24  (1·5) | .. | 7  (0·9) | .. | 48  (2·9) |
